# The development and features of the Spanish prehospital advanced triage method (META) for mass casualty incidents

**DOI:** 10.1186/s13049-016-0255-y

**Published:** 2016-04-29

**Authors:** Pedro Arcos González, Rafael Castro Delgado, Tatiana Cuartas Alvarez, Gracia Garijo Gonzalo, Carlos Martinez Monzon, Nieves Pelaez Corres, Alberto Rodriguez Soler, Fernando Turegano Fuentes

**Affiliations:** Unit for Research in Emergency and Disaster, Department of Medicine, Universidad de Oviedo, Oviedo, Spain; SAMU-Asturias, Oviedo, Spain; Emergencias Osakidetza, País Vasco, Spain; Hospital de Martorell. Sistema Emergencies Médiques, Barcelona, Spain; School of Nursing Nuestra Señora de La Candelaria, Universidad de La Laguna, Santa Cruz de Tenerife, Spain; Hospital Universitario Gregorio Marañón, Madrid, Spain

## Abstract

This text describes the process of development of the new Spanish Prehospital Advanced Triage Method (META) and explain its main features and contribution to prehospital triage systems in mass casualty incidents. The triage META is based in the Advanced Trauma Life Support (ATLS) protocols, patient’s anatomical injuries and mechanism of injury. It is a triage method with four stages including early identification of patients with severe trauma that would benefit from a rapid evacuation to a surgical facility and introduces a new patient flow by-passing the advanced medical post to improve evacuation. The stages of triage META are: I) Stabilization triage that classifies patients according to severity to set priorities for initial emergency treatment; II) Identifying patients requiring urgent surgical treatment, this is done at the same time than stage I and creates a new flow of patients with high priority for evacuation; III) Implementation of Advanced Trauma Life Support protocols to patients previously classified according to stablished priority; and IV) Evacuation triage, stablishing evacuation priorities in case of lacks of appropriate transport resources. The triage META is to be applied only by prehospital providers with advanced knowledge and training in advanced trauma life support care and has been designed to be implemented as prehospital procedure in mass casualty incidents (MCI).

## Background

Mass casualty incidents (MCI) are defined by World Health Organization as events which generate more patients at one time than locally available resources can manage using routine procedures [1]. A recent population based epidemiological study has identified more MCI than expected [2], and this should be taken into consideration when planning the response. This means that special procedures must be used in these situations in order to save as many lives as possible. One of these procedures is triage [3], defined as classification of patients in various categories according to severity and prognosis, to determine the priority of treatment and evacuation [4]. A number of studies have been conducted to try to define adequate specificity and sensitivity of a suitable triage method to be used in the prehospital scene [5] and a systematic review concluded that “there is a lack of scientific evidence about the effects of validated pre-hospital triage systems and about the effects of using the same triage system in two or more settings of the Emergency Medical Services (EMS)” [6]. Some triage methods have demonstrated good performance in training exercises [7, 8] but not in real incidents [9].

Most of these triage methods are based in basic life support techniques and have been designed to be applied by rescue teams and firemen [10]. The specific and complex development of EMS worldwide [11, 12] have made necessary to develop new triage methods adapted to advanced medical care in the prehospital setting in order to take advantage of medical knowledge that advanced medical teams can perform on the field [13]. In a MCI basic triage methods can underestimate severity of injured people and can lead to an unappropiate prehospital care or even overestimate severity which could lead to overuse of resources for patients that don’t need them [14]. Some recently developed triage methods have tried to improve those aspects [15]. A relevant element to be improved in advanced triage methods is early detection of severe injured patients that could benefit from rapid transport to a surgical facility, instead of delaying transport due to overhealmed resources in the prehospital setting.

The aim of this paper is to present the development process and design of the Spanish prehospital advanced triage method (META, Spanish acronym for *Modelo Extrahospitalario de Triage Avanzado*) as well as its main features and field operating mode in a mass casualty incident. It should be noted that META is a method of *advanced* triage type to be used only by advanced prehospital providers with adequate training and education in advanced trauma life support protocols and techniques.

## Methods

The development process of the META was made in several stages: in the first stage we performed an extensive literature review of the currently available prehospital triage methods, their features and contextual healthcare system in which they were designed in order to identify and list all parameters that could be potentially used in the prehospital classification of victims (triage) during a MCI.

During the second phase of the study the degree of perceived usefulness and feasibility of use in the setting of an MCI were analyzed for each of the selected parameters by a sample of health professionals of the Spanish healthcare system. To do this a survey was designed for doctors and nurses working in hospital emergency departments and prehospital emergency care systems in which they were asked to evaluate three dimensions for each parameter: (i) Ability to predict the patient’s vital risk, (ii) Ability to prioritize patient evacuation and (iii) Feasibility of use of the parameter in the prehospital setting in case of MCI. To assess the relevance of each parameter a numerical scale from one (irrelevant) to ten (maximum relevance) was used in each of the three dimensions.

Once studied the degree of perceived usefulness and feasibility of use of each parameter during the third phase of the study, a wide panel of experts from different backgrounds (doctors, nurses, prehospital care staff, hospital emergency room and surgical trauma care staff) finally decided the parameters to be included in the triage method and sequence of application of each parameter during the triage process.

## Results

Forty-five parameters were identified in literature review as potentially usable in an MCI advanced triage method. Eight out of those 45 identified parameters were *anatomical type* parameters, 19 were *physiological type* parameters, nine were parameters *related to the kind of injury* and nine were *related to other conditions or circumstances of the victim or the MCI* different of those already mentioned. Appendix shows the complete list of 45 identified parameters.

**Fig. 1:**
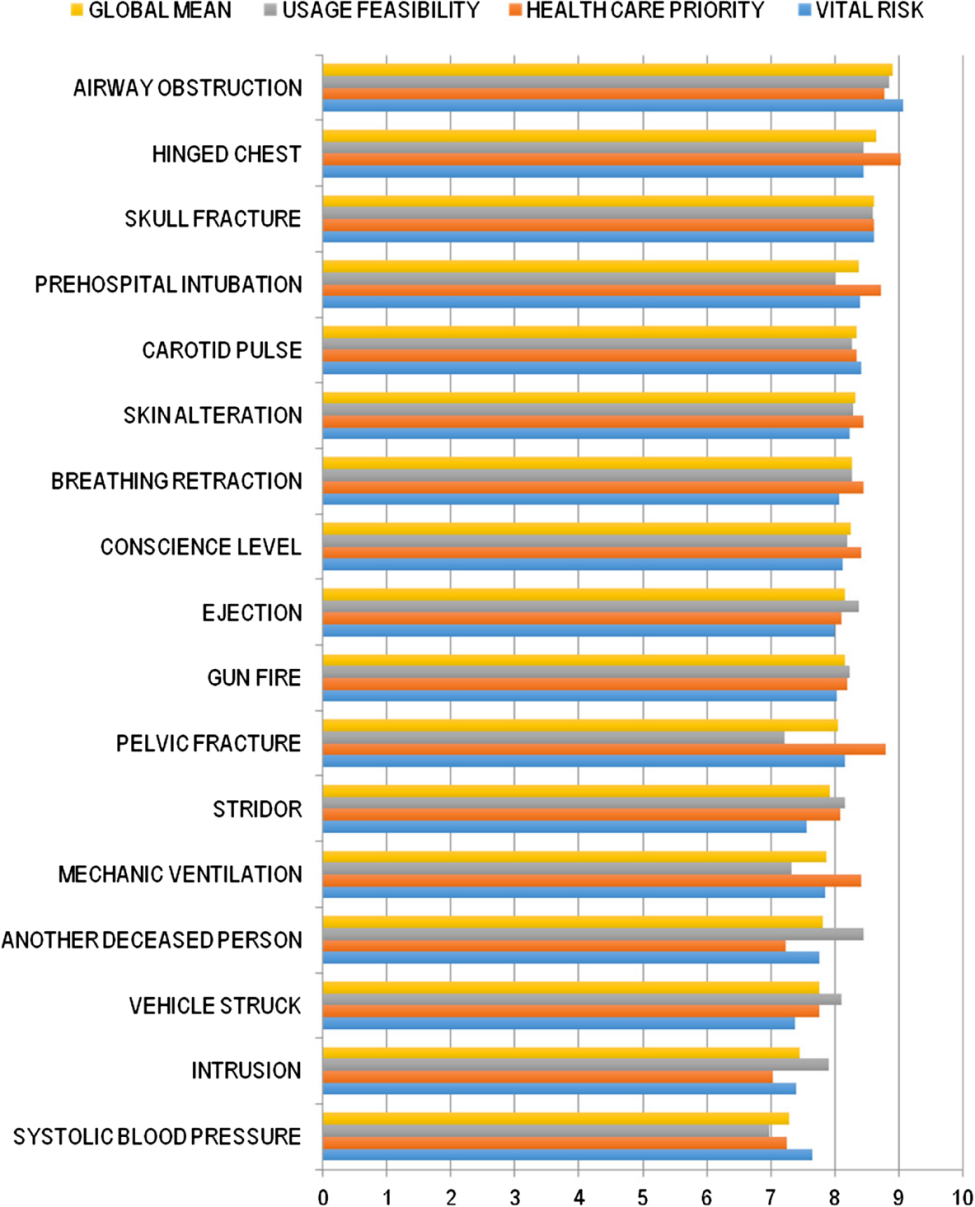
Parameters perceived as significantly relevant (*p* < 0.05) to be included in a system of triage

Seventeen out of the 45 parameters studied were found to be significantly relevant (*p* <0.05) for potential inclusion in the META. Table 1 and Fig. 1 show the scores obtained for the three dimensions studied for each parameter, as well as the overall mean. Ten of them, most of them with higher scores, were related to severe trauma patient clinical evaluation and advanced trauma life support (ATLS). The rest were related to mechanism of injury and anatomical lesions. Taking in consideration these results, the panel of experts organized the selected parameters to design the final triage method which is mainly based in the ATLS protocols and in severe trauma patient field triage to detect potential surgical life threatening injuries.

**Table 1:** Mean scores of parameters perceived as significantly relevant (*p* < 0.05) to be included in a system of triage in an scale from one to ten

Parameter	Vital risk	Health care priority	Usage feasibility	Global mean
Systolic blood pressure	7,646153846	7,246153846	6,98461538	7,292307691
Intrusion	7,4	7,030769231	7,90769231	7,446153847
Vehicle struck	7,384615385	7,769230769	8,10769231	7,753846155
Another deceased person	7,753846154	7,230769231	8,44615385	7,810256412
Mechanic ventilation	7,861538462	8,415384615	7,32307692	7,866666666
Stridor	7,553846154	8,092307692	8,15384615	7,933333332
Pelvic fracture	8,169230769	8,8	7,21538462	8,061538463
Gun fire	8,030769231	8,2	8,23076923	8,153846154
Ejection	8	8,107692308	8,38461538	8,164102563
Conscience level	8,123076923	8,415384615	8,2	8,246153846
Breathing retraction	8,076923077	8,461538462	8,27692308	8,271794873
Skin alteration	8,230769231	8,446153846	8,29230769	8,323076922
Carotid pulse	8,415384615	8,338461538	8,27692308	8,343589744
Prehospital intubation	8,4	8,723076923	8,01538462	8,379487181
Skull fracture	8,615384615	8,615384615	8,6	8,61025641
Hinged chest	8,461538462	9,030769231	8,44615385	8,646153848
AIRWAY OBSTRUCTION	9,076923077	8,784615385	8,84615385	8,902564104

**Fig. 2:**
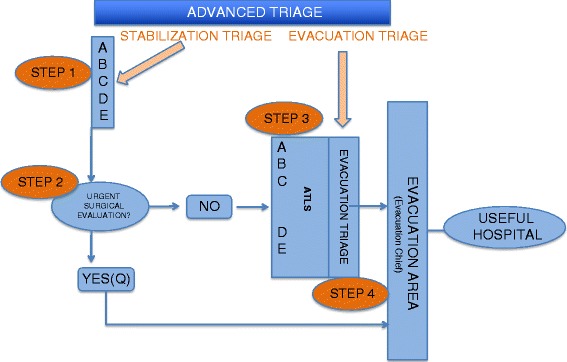
Main steps in META triage model

META triage is to be implemented during the medical prehospital response to an MCI and has four stages: 1) Stabilization triage, 2) Identification of need of urgent surgical care, 3) Advanced trauma life support techniques and 4) Evacuation triage (Fig. 2).

1. Stabilization triage: at this stage patients are initially evaluated using the advanced trauma life support protocols and every patient with actual or potential risk for airway, breathing or circulation is classified as red. Patients with single neurological disability or in need of hospital evaluation after brief exposition are classified as yellow. The rest of the patients are classified as green. At this stage only basic life saving interventions are performed like basic airway opening and haemorrhage control with pressure or tourniquet [16] (Fig. 3).

**Fig. 3:**
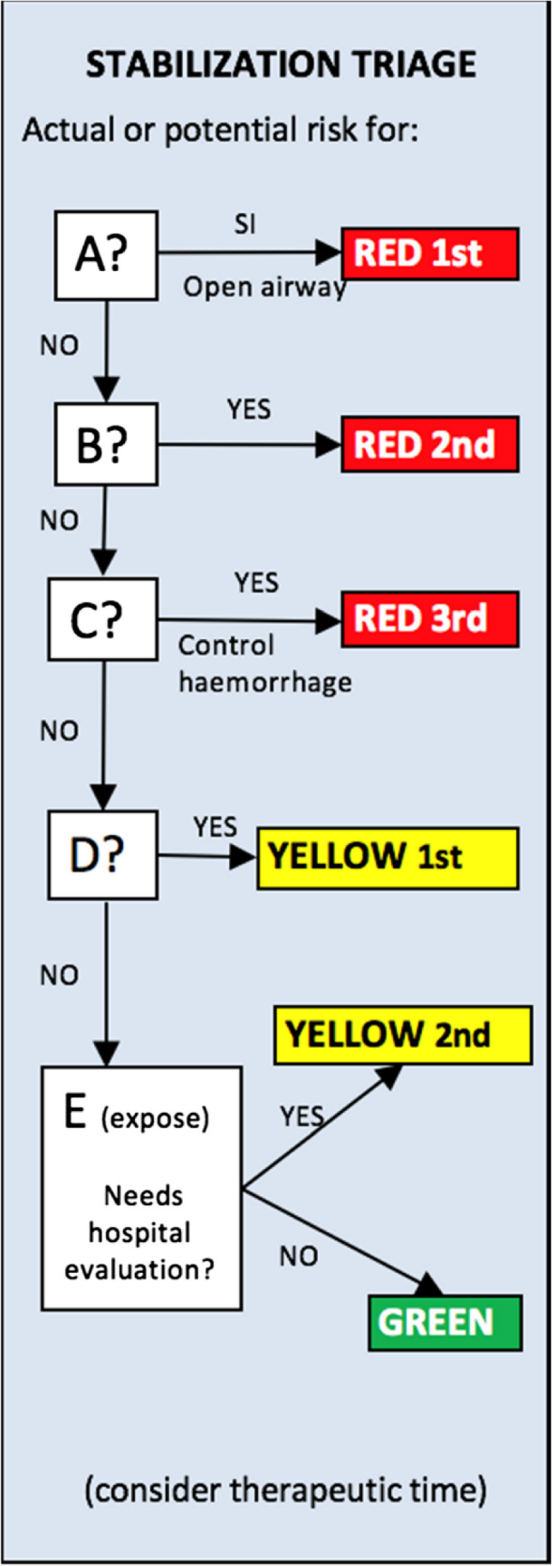
Stabilization triage
2. Identification of need of urgent surgical care. The aim of this stage (applied at the same time as first stage) is to identify patients who do not benefit of complex prehospital care and needs rapid transport to a surgical facility. This creates a new flow of patients that will by-pass advanced medical post and will be directed to evacuation area with a minimum acceptable care. For these purpose we used the Guidelines for field triage of injured patients developed in 2011 by the National Expert Panel of Field Triage [17]. These recommendations were adapted by the panel of experts to be applied in EMS with advanced resources. Final recommendations in this stage are: a) All penetrating injuries to head, neck, torso and extremities proximal to elbow or knee, b) Open pelvic fracture, c) Closed pelvic fracture with mechanical or haemodinamical instability and d) Blunt torso trauma with haemodinamic instability.
3. Advanced Trauma Life Support. All patients, once classified will be treated following advanced trauma life support protocols [18]. Red patients from first stage will be treated first, then yellow and finally green.
4. Evacuation triage. Once we have treated out of hospital emergencies on the field, we have to decide, in a scarced resources situation, which patient needs to be evacuated first. The first ones will be those with urgent surgical care need that have not been detected prior to evacuation. Then, we have define a new cathegory as “hight priority criteria” for those who have severe injury with haemodynamic or respiratory instability and one of these: systolic blood pressure under 110 mmHg [19], motor Glasgow coma score under six [20], intubation or explosion in confined space [21]. Patients with airway, breathing or circulation compromise not solved with high priority criteria will be first evacuated, then those with ABC compromise not solved but without high priority criteria. Then red patients with solved ABC compromise. All these patients will have red tags [22]. Next will be patients with single neurological disability, and finally those needing hospital evaluation but without any of the mentioned situations (Fig. 4). The full META triage model is represented in Fig. 5.

**Fig. 4:**
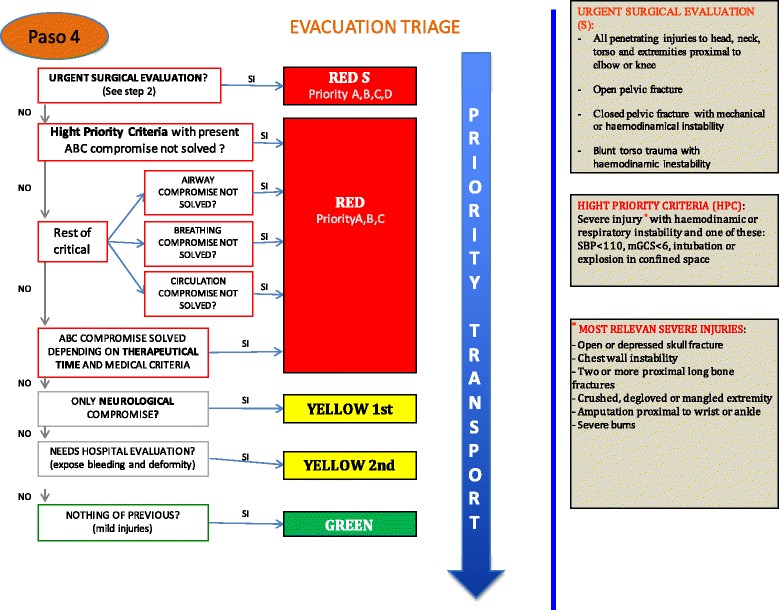
Evacuation triage

**Fig. 5:**
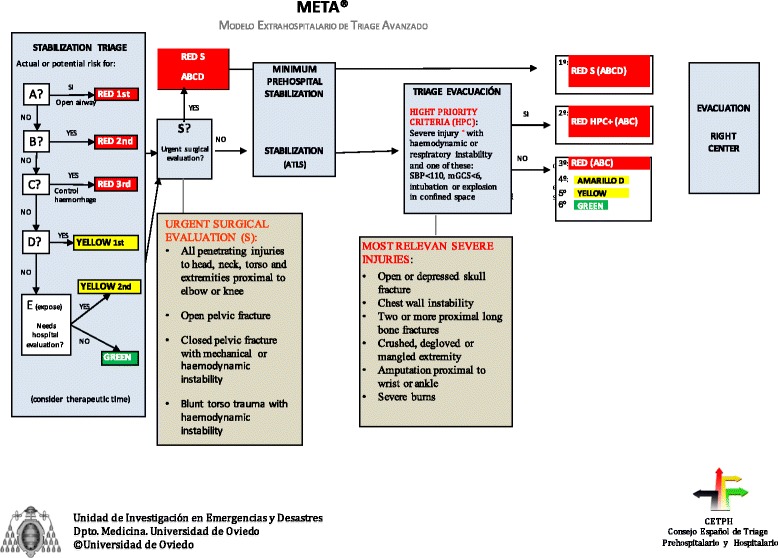
Complete META triage model

## Discusion

The selection of the variables identified as potentially be incorporated to an advanced triage method is based primarily on well-known aspects of the clinical approach to prehospital treatment of patients with severe trauma, as well as in physiological, and anatomical aspects and the mechanism of injury. Once evaluated by doctors and nurses, parameters with higher scores are those related to physiological aspects. We decided to design a method based in the ATLS protocol but with an anatomical component that creates a new high priority category for those patients that benefit from rapid transport to a surgical facility.

This new model of triage would be useful specially for EMS staffed by doctors or nurses, but also for EMS based in paramedics with an advanced education, knowledge and skills in the management of patients with acute severe trauma. Most of the well known triage methods do not fulfil the principles of the advanced trauma life support. In the above triage methods [23], for example, if a patient is able to walk will be classified as mild patient (green), but most doctors and nurses know that a patient with severe burns can walk, or even a patient in shock due to abdominal bleeding can also do it in the early stages of shock; both are typical examples of undertriage. Another example would be a patient with a respiratory rate of 35 per minute caused by a state of anxiety and should not be classified as severe. These two are typical examples of patients benefiting from using a more accurate method of triage to be able to early identify circumstances that may threaten their lives or to identify those patients who do not even need a hospital evaluation. These are typical cases in wich the triage META would provide a more accurate triage in a MCI. Also the fact that the design of META has taken into account the perception of doctors and nurses on different aspects of the parameters included suggests that the method is better received by advanced prehospital care providers. On the other hand, the fact that the organization of triage META is done in different stages makes it easy to incorporate into the usual procedures in MCI. The first stage on META helps us to detect life-threatening injuries, gives us an initial idea for the organization and helps us to prioritize patients. We only apply the basic life-saving techiques to keep patient alive waiting for the next stage, and at the same time we apply second stage to identify those injuries that threaten the patient’s life and who benefit from rapid transport to surgical facility.

In a MCI with overwhelmed healthcare resources the flow of patients at different stages of the medical response is often slow. This means that patient transportation may be delayed and for some patients this could be life threatening [24]. This is one of the reasons why we have created a second stage to identify those patients who have a high priority for evacuation so as not to have to go through all medical posts but go directly from the triage area to the evacuation area with minimum acceptable care. Third stage is useful to apply advanced trauma life support techniques according to patients needs and means that priority may change. Most advanced techniques are performed in this stage for advanced life support teams who have to decide, according to the needs and available resources, which are the most feasible techniques according to the circumstances.

Once a patient is treated or stabilized should go to the evacuation point. In this point we go to stage four for evacuation triage. This last triage allows us to use available transport resources in the best possible way. In case of lack of transport resources patients classified as red have the highest priority for evacuation. These “high priority criteria” allows us to distinguish different priorities among patients classified as red and are based in a combination of clinical elements and injury mechanisms in order to improve evacuation of severe patients. This new method of advanced triage is highly adaptable to future scientific findings related to prehospital care and organization of MCI.

One of the strengths of triage META is that has been developed taking into account the perception of emergency services staff on the factors to be considered in a method of triage and this is an important aspect that influences the success in implementation in daily work. Also the varied background of the expert panel has made possible a broad discussion on the different aspects and perceptions of the management of trauma care in the prehospital heathcare of MCI. Triage research has many limitations and is very difficult to fulfil the best evidence recommendations [25]. Our method of triage has been developed in a country with a specific health system, which is the type of European public health systems with universal coverage. It would be necessary to consider how its applicability is affected in countries with characteristics very different from ours context.

## Conclusion

Triage META is a model of advanced prehospital triage and is a tool to be used by doctors and nurses trained as providers of advanced trauma life support, but also by paramedics with advanced education, knowledge and skills in management of patients with severe acute trauma. It can be implemented into MCI procedures and one of the main contributions is the early detection of severe surgical patients that benefit from rapid transport to a surgical facility. This mean that two flows of patients are needed in order to avoid delays of transport in these patients.

## Appendix

### Appendix: Parameters identified in literature review as potentially usable in an MCI advanced triage method

**Anatomical Paramethers**

Amputation proximal to the wrist or ankle

Tear or crushing of limbs

Open or depressed skull fracture

Pelvic fracture

Proximal fractures of two or more long bones

Penetrating wound

Contused wound

Flail chest

**Physiological paramethers**

Heart Rate

Respiratory rate

Prehospital intubation

Level of consciousness

Paralysis

Carotid Pulse

Radial pulse

Ventilation

Airway obstruction

Oxygen saturation

Systolic blood pressure

Staring

Pediatric Evaluation Triangle

Spotted, pallor or cyanosis of the skin

Respiratory distress

Stridor, grunting or breathing sound

Speech disorder or crying

Flaring nostrils

Interactivity

**Mechanism of injury**

Knive njury

Firearm

Outrage

Fall

Rescue time more than 20 minutes

Ejected from vehicle

Presence of a died person in the same vehicle

Vehicle Intrusion

Motorbike accident

**Others**

Medical Criteria

Overtriage if case of doubt regarding severity of the patient

Age under 15 or over 55

Pregnant more tan 20 weeks

Severe kidney disease

Limb injury time dependant

Burns

Sex

Haemostatiuc disorder or anticoagulants treatment
